# Enhanced Viability and Anti-rotavirus Effect of *Bifidobacterium longum* and *Lactobacillus plantarum* in Combination With *Chlorella sorokiniana* in a Dairy Product

**DOI:** 10.3389/fmicb.2020.00875

**Published:** 2020-05-12

**Authors:** Servando Cantú-Bernal, Maribel Domínguez-Gámez, Ivana Medina-Peraza, Elizama Aros-Uzarraga, Noé Ontiveros, Lilian Flores-Mendoza, Ricardo Gomez-Flores, Patricia Tamez-Guerra, Guadalupe González-Ochoa

**Affiliations:** ^1^Laboratorio de Inmunología y Virología, Departamento de Microbiología e Inmunología, Facultad de Ciencias Biológicas, Universidad Autónoma de Nuevo León, San Nicolás de los Garza, Mexico; ^2^Laboratorio de Microbiología e Inmunología, Departamento de Ciencias Químico Biológicas y Agropecuarias, Universidad de Sonora, Sonora, Mexico

**Keywords:** microalga, probiotic, flan, shelf-life, metabolites, rotavirus

## Abstract

Microalgae and probiotics such as *Bifidobacterium* and *Lactobacillus* genera are associated with human beneficial effects. The aim of this study was to evaluate the activity of *Chlorella sorokiniana* on *Bifidobacterium longum* and *Lactobacillus plantarum* viability in a dairy product (flan) and its microbial effect against rotavirus, which is one of the major diarrhea-causing pathogens worldwide. Microalge were isolated from a Mexican river and characterized by molecular tools. Their prebiotic activity was evaluated by determining *Bifidobacterium longum* and *Lactobacillus plantarum* shelf-life after incorporation in the food matrix. In addition, HT-29 cells were infected with rotavirus Wa and treated with 1 × 10^9^ CFU/mL *L. plantarum* and *B. longum* metabolites alone or in combination with 1 × 10^9^ cells/mL *Chlorella sorokiniana*; viral titers in probiotics- and/or microalgae-treated cells were evaluated for antiviral activity. Results indicated that *C. sorokiniana* not only significantly (*p* < 0.05) improved *L. plantarum* and *B. longum* viability in flan, but also increased their antiviral activity; potent anti-rotavirus effect of *C. sorokiniana* alone was observed. Although more studies are needed, results suggest that incorporation of this microalga into a dairy product confers enhanced viability and antiviral effects, which indicates that *C. sorokiniana* might be used as an ingredient to design products with additional health benefits.

## Introduction

The microalga *Chlorella* spp. is an important source of bioactive compounds such as proteins, fatty acids, carotenoids, sulfated polysaccharides, vitamins, and other natural bioactive molecules. This microalga is an important ingredient in functional foods because of its nutritional and pharmaceutical properties (de Morais et al., 2015; Silva et al., 2019). In addition, *Chlorella* spp. extracts have shown potential to modulate human immune response, accelerate dioxin elimination, prevent stress-induced ulcers, and decrease high-fat-diet-induced dyslipidemia (Ciliberti et al., 2019). Furthermore, microalga extracts incorporation into food triggers an array of metabolic and immunological processes in human and monogastric animal intestines stressed by enteric bacterial pathogens (Hulst et al., 2019). Notably, microalga sulfated polysaccharides have been associated with blocking viral entry to cells, thus avoiding infection (Guedes et al., 2011).

Probiotics are often used to prevent enteric diseases (Sales-Campos et al., 2019) and decrease diarrhea severity, days with symptoms, and reinfections (Huang et al., 2002). Particularly, *Bifidobacterium* and *Lactobacillus* genera are the most commonly associated probiotics with beneficial effects for humans, mainly against enteric infections, such as the one caused by rotavirus (Gonzalez-Ochoa et al., 2017; Park et al., 2017). These probiotics and their metabolites modulate the cytokine milieu, stimulated by the gut-associated lymphoid tissue and improves intestinal barrier function against pathogens by promoting an efficient immune response (Vlasova et al., 2016; Tang and Lu, 2019).

Although probiotics have shown beneficial effects on human health, they are strain-, dose-, and viability-dependent. For instance, in probiotic fermented milks production, the viability of probiotics is lost during the fermentation process and storage at cold (refrigerator) conditions (Terpou et al., 2019). However, probiotics must be viable and abundant (10^6^ CFU/gr) at the time of food product consumption to obtain the health benefits associated with these microorganisms (Terpou et al., 2019). Probiotics effectiveness would be improved by incorporating prebiotics into the food matrix; they produce compounds that stimulate probiotics growth and function (Beheshtipour et al., 2013). The aim of the present study was to evaluate the effect of *C. sorokiniana* to improve the viability of *Bifidobacterium longum* and *Lactobacillus plantarum* in a stored dairy food product and determine its antimicrobial effect against rotavirus, which is one of the main causing-diarrhea pathogens worldwide.

## Materials and Methods

### Probiotics

The probiotics strains *Lactobacillus plantarum* (ATCC LP299v) and *Bifidobacterium longum* (ATCC 15707) were kindly provided by Porfiria Barrón from the Laboratorio de Biología Celular of Facultad de Ciencias Biológicas at Universidad Autónoma de Nuevo León, México. They were grown on MPT medium as previously described (Mendoza-Flores, 2014; González and Quiñones-Gutiérrez, 2018). This medium contains 2.5 g yeast extract, 0.50 g sodium chloride, 0.5 g L-cysteine hydrochloride, 0.05 g ascorbic acid, 0.25 g potassium phosphate dibasic, 0.15 g potassium phosphate monobasic, 0.124 mg ferric ammonium citrate, 10 g casein digest peptone, and 5.0 g glucose (González and Quiñones-Gutiérrez, 2018). Microbial growth kinetics was determined by turbidimetry; once the exponential phase was identified, colony-forming units per milliliter (CFU/mL) were calculated by serial dilutions on agar plates and the bacterial inoculum was adjusted to 1 × 10^6^ or 1 × 10^9^ CFU/mL, depending on the assay.

### Cells

Human colon cancer (HT-29; ATCC^®^ HTB-38^TM^) and African green monkey kidney (MA104; ATCC^®^ CRL-2378^TM^) cell lines were used in this study. They were grown in RPMI medium (Gibco/Life Technologies, Grand Island, NY, United States) supplemented with 5% of fetal bovine serum (FBS; Mediatech, Herndon, VA, United States), 2 mM L-glutamine, and 1% antibiotic and antimycotic solution (Caisson Laboratories, North Logan, UT, United States), and incubated at 37°C in 5% CO_2_, until confluence. Cells were then harvested using PBS and 0.25% trypsin (Mediatech, Herndon, VA, United States) at 37°C, until detachment from the culture flask, and transferred to 6-well plates for probiotics, microalgae, and rotavirus assays or 96-well plates for rotavirus microtitration. Probiotics and microalga cytotoxicity to HT-29 and MA014 cells was determined by the MTT reduction assay, as previously described (Mosmann, 1983).

### Rotavirus Strains

The rotavirus strain Wa was kindly provided by Dra. Susana López from the Instituto de Biotecnología at Universidad Nacional Autónoma de México. Viral particles were propagated in MA104 cells and activated with 10 μg/mL of trypsin, incubated at 37°C in 5% CO_2_ for 1 h, the inoculum replaced by RPMI medium without FBS, and incubated at 37°C in 5% CO_2_, until cell lysis. Viral titers were calculated as focus forming units per milliliter (FFU/mL), using an immunochemistry assay protocol (Chasey, 1980). In brief, lysates from virus propagation or cells infected with rotavirus and treated with probiotics and the microalga were used to infect MA104 in 96-well plates; after 14 h of incubation, cells were fixed with a cold acetone-PBS solution and incubated for 45 min at room temperature. Monolayer was then washed twice with PBS, primary anti-rotavirus antibodies (Invitrogen, Carlsbad, CA, United States) were added, and cells incubated 1 h at 37°C and washed. Next, horseradish peroxidase (HRP)-anti-sheep IgG conjugate (Invitrogen, Carlsbad, CA, United States) was added to the cells and incubated 1 h at 37°C, followed by the addition of the substrate aminoethylcarbazole (0.64 mg/mL; Sigma-Aldrich, St. Louis, MO, United States), prepared in dimethylformamide (Sigma-Aldrich) with acetate buffer (30 mM sodium acetate and 12 mM acetic acid), pH 5.0, plus 0.36% hydrogen peroxide. Infected cells were counted using optical microscopy and identified by their brown color, indicating presence of viral antigens. FFU/mL were calculated using the following formula: FFU/mL = 20 × (microscope objective) × 5.5 (well diameter) × average number of focus (duplicate, 100–200 focus count/well) × dilution (focus count). Rotavirus multiplicity of infection (MOI) was 0.01 in each assay.

### Microalgae Growth Conditions and Characterization

Microalgae were isolated in the river San Juan Cadereyta in Nuevo León, Mexico (Reyna-Martinez et al., 2018). They were grown in LC nutrient solution (5 mM KNO_3_, 1 mM KH_2_PO_4_, 2 mM MgSO_4_⋅7H_2_O, 6.25 mM Ca(NO_3_)_2_⋅4H_2_O, 46 μM H_3_BO_3_, 9.15 μM MnCl_2_⋅4H_2_O, 765 nM ZnSO_4_⋅7H_2_O, 320 nM CuSO_4_⋅5H_2_O, 15 nM (NH_4_)6Mo7O_24_⋅4H_2_O, 20 μM FeSO_4_⋅7H_2_O, and 20 μM Na_2_EDTA) at 25°C and 120 rpm with continuous light at 1,400 lumens for 12 days (López-Chuken et al., 2010). Microalgae were microscopically classified as the green microalga *Chlorella* spp. and was genetically classified by molecular tools as follows: 40 mg of biomass in 1 mL of PBS were frozen with liquid nitrogen, homogenized at 8,000 rpm for 1 min, followed by DNA extraction, using the Purelink Genomic DNA kit (Invitrogen, Carlsbad, CA, United States). Purified DNA was used to amplify the 18S ribosomal RNA gen, using the primers P2F 5′-GGCTCATTAAATCAGTTATAG-3′/P2R 5′-C CTTGTTACGA(C/T)TTCTCCTTC-3′ (Lee and Hur, 2012). PCR amplicon (1700 bp) was purified with the Wizard SV Gel and PCR *clean-up system Kit* (Promega, Madison, WI, United States) and sequenced twice by the dideoxynucleotide chain termination method, using an ABI Prism BigDye terminator cycle sequencing ready reaction kit (Applied Biosystems, Foster City, CA, United States). The sequence of the isolated microalgae was submitted to the GenBank database with the access number MN011866. This was analyzed and compared with other sequences reported in GenBank database; phylogenetic relationships between strains were reconstructed using the maximum likelihood method with 1,000 replicates using MEGA X software (Kumar et al., 2018).

### Proximate Composition of Microalgae Biomass

Association of Official Analytical Chemist (AOAC) methods were followed in detail. In brief, moisture was evaluated by drying the microalgae at 105°C for 24 h (method 925.09B); crude protein was assessed using the micro-Kjeldahl assay (method 960.52); lipids were evaluated using a Soxhlet apparatus and petroleum ether as solvent (method 920.39C); ashes were calculated after incineration at 550°C (method 923.03), and carbohydrates by difference of the other components (AOAC, 1999). The results were expressed in mg/g on a dry-matter basis. All evaluations were determined in triplicate.

### Probiotics and Microalgae Combination in a Dairy Product

Combinations were made with probiotics at 1 × 10^9^ CFU/mL and *Chlorella* spp. at 1 × 10^6^ cells/mL or 1 × 10^9^ cells/mL in 2 g of a dairy product such as flan and stored at 4°C. Probiotics viability was monitored every 72 or 96 h of storage as follows: 1 g of flan containing the probiotic and/or microalgae was disrupted in PBS and diluted to calculate CFU/g. Microalgae were cultured independently from bacteria, thus, live microalgae were combined with probiotics. Assays were designed to take up to 34 days; nevertheless, results provided in figures indicated different treatment times, because experiments were ended once probiotic counts were lower than 10^6^ UFC/gr.

### Probiotics and Microalgae Combination Against Rotavirus

HT-29 cells were infected with rotavirus Wa (MOI 0.01), incubated 1 h at 37°C in 5% CO_2_, and treated with 2 mL of probiotic metabolites (*Lactobacillus plantarum* and/or *Bifidobacterium longum* at 1 × 10^9^ CFU/mL) and/or *Chlorella sorokiniana* (1 × 10^9^ CFU/mL) in RPMI medium without FBS, followed by incubation at 37°C in 5% CO_2_ for 24 h. After this, cells were stored at −20°C, until viral titration by immunochemistry.

### Statistical Analysis

Data were reported as mean ± SD of triplicate determinations from three independent experiments. Statistical analysis was determined by two-way ANOVA and Fisher’s LSD multiple comparisons or Kruskal-Wallis and Dunn ìs multiple comparisons, using GraphPad Prism 7 (GraphPad Software Inc., San Diego, CA, United States).

## Results

### Phenotypic, Molecular, and Proximate Analyses of Microalgae

**FIGURE 1:**
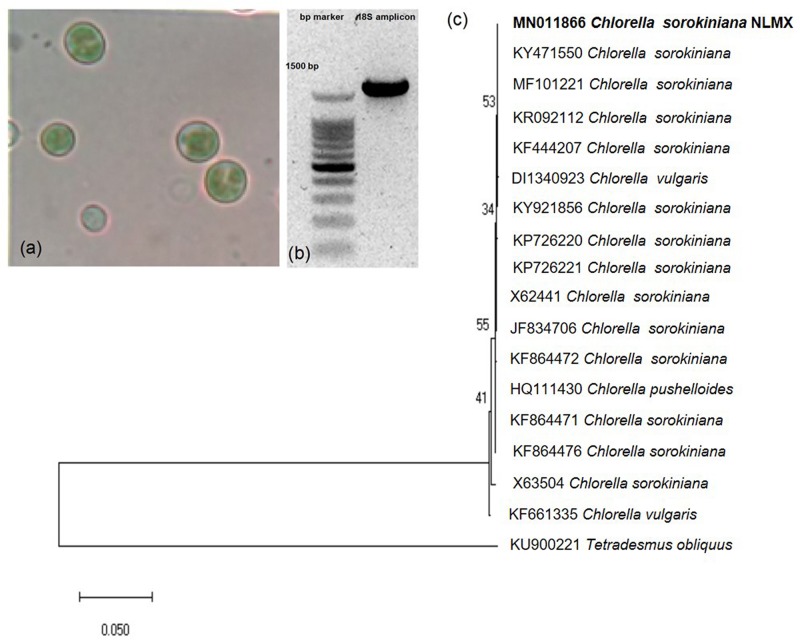
Microalgae phenotypic and molecular characterization. **(a)** Microscopic characteristics of *Chlorella* spp. **(b)** Partial amplification of *Chlorella* spp. 18S ribosomal RNA gene, **(c)** Phylogenetic analysis of 18S ribosomal RNA amplicon which clustered with previously reported *Chlorella sorokiniana* sequences. The evolutionary history was inferred by using the Maximum Likelihood method and Tamura-Nei model. The tree is drawn to scale, with branch lengths measured in the number of substitutions per site. This analysis involved 18 nucleotide sequences. There were 1,596 positions in the final dataset. Evolutionary analyses were conducted in MEGA X (Kumar et al., 2018).

Observed microalga characteristics indicated their correspondence with the genera *Chlorella* spp. (Figure 1a). The homologous sequence (99.7–100%) of the 18S ribosomal RNA gene (Figure 1b) and phylogenetic (Figure 1c) analyses revealed the presence of the green microalga *Chlorella sorokiniana.* Biomass analysis showed that this microalga was constituted on a dry-matter basis of 45.54% (455.4 mg/g) ash, 30.77% (307.7 mg/g) proteins, 1.15% (11.5 mg/g) lipids, and 22.53% (225.3 mg/g) carbohydrates (Table 1).

**TABLE 1:** Proximate composition of *Chlorella sorokiniana* biomass.

Components	Content mg/g
Protein	307.7 (±24)
Carbohydrates	225.3 (±28)
Lipids	11.5 (±3.3)
Ash	455.4 (±43)
Moisture	122 (±6)

### Incorporation of *Chlorella sorokiniana* Into a Dairy Product Increases Probiotics Viability

**FIGURE 2:**
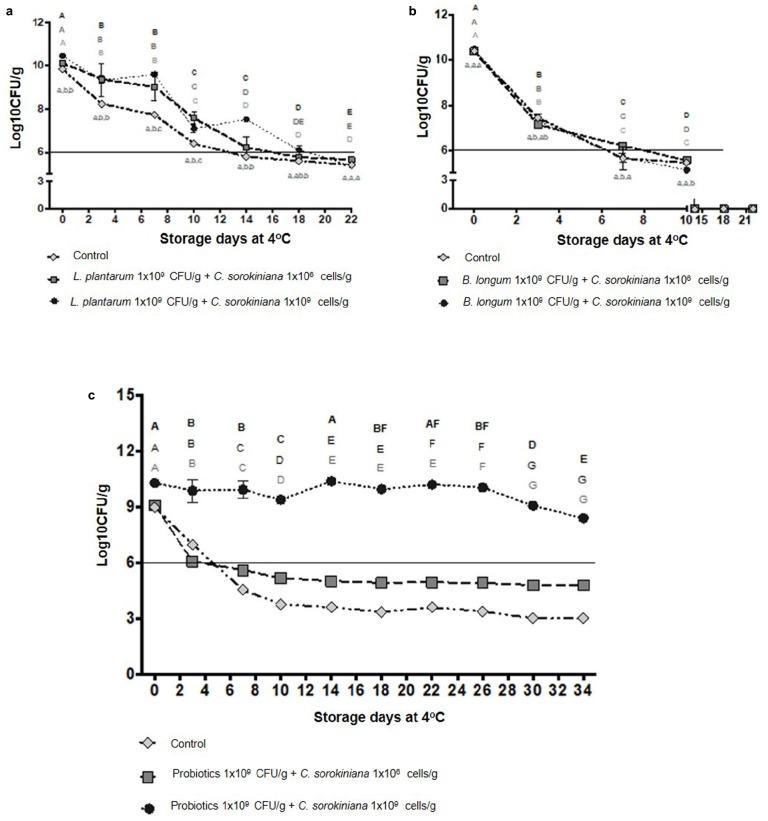
Viability of *Lactobacillus plantarum* and *Bifidobacterium longum* in combination with *Chlorella sorokiniana* in a dairy product. **(a)** *L. plantarum* viability (days) alone and in combination with *C. sorokiniana*. **(b)** *B. longum* viability (days) alone and in combination with *C. sorokiniana*. **(c)** *L. plantarum* and *B. longum* viability (days) with and without *C. sorokiniana*. Lowercase letters indicate statistical significances between treatments at a specific time. Capital letters indicate statistical significances in treatment over time. Gray letters represent the control, black letters indicate *L. plantarum* or *B. longum* (1 × 10^9^ CFU/g) + *C. sorokiniana* (1 × 10^6^ Cells/g), and bold black represent *L. planatrum* or *B. longum* (1 × 10^9^ CFU/g) + *C. sorokiniana* (1 × 10^9^ Cells/g). Data were analyzed by two-way ANOVA with subsequent Fisher ìs LSD test using GraphPad Prism 7.0.

The probiotics *Lactobacillus plantarum* and/or *Bifidobacterium longum* in combination with *Chlorella sorokiniana* were added to flan and stored at 4°C and probiotics viability evaluated every 72 or 96 h. *L. plantarum* and *B. longum* showed viability up to 1 × 10^6^ CFU/g at 10 and 7 days, respectively, whereas addition of *C. sorokiniana* (1 × 10^9^ cells/mL) into the dairy product extended *L. plantarum* viability up to 18 days (*p* < 0.05) (Figure 2a); in contrast, it did not prolong that of *B. longum* (Figure 2b). On the other hand, *L. plantarum, B. longum*, and *C. sorokiniana* combination in flan showed longer viability (>34 days) than that of probiotic combinations without microalgae (*p* < 0.05) (Figure 2c).

### Probiotic Metabolites and *C. sorokiniana* Reduce Rotavirus Infectivity

**FIGURE 3:**
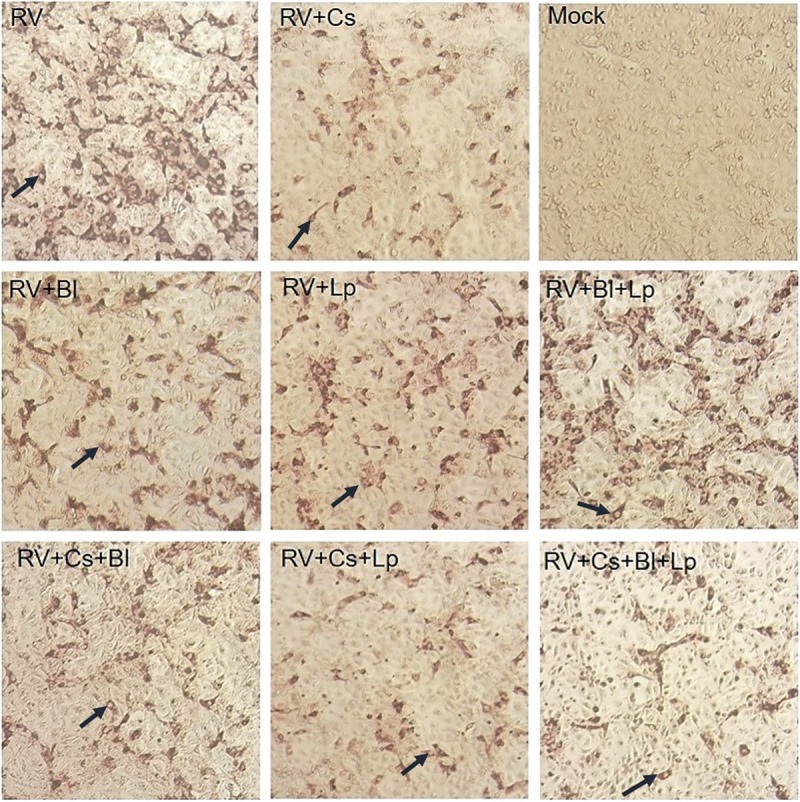
Rotavirus focus forming units in HT-29 cells treated with combination of probiotics and/or *Chlorella sorokiniana.* RV, Rotavirus; Bl, *Bifidobacterium longum*; Lp, *Lactobacillus plantarum;* Cs, *Chlorella sorokiniana.* Arrows indicate units forming focus in each assay.

**FIGURE 4:**
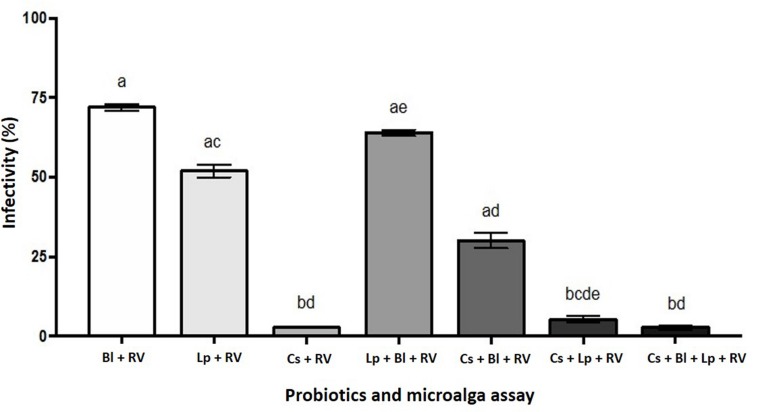
*B. longum*, *L. plantarum*, and/or *C. sorokiniana* metabolites against rotavirus. Assays were performed in HT-29 cells, whereas viral titers were determined in MA104 cells. Viral titers of each assay were calculated as FFU/mL; viral infectivity percentage was estimated considering rotavirus titers in infected cells without any treatment as 100% infectivity. RV, rotavirus; Bl, *Bifidobacterium longum*; Lp, *Lactobacillus plantarum*; Cs, *Chlorella sorokiniana.* Different letters indicate statistical significances among treatments. Gray letters represent the control, black letters indicate probiotics (1 × 10^9^) + *C. sorokiniana* (1 × 10^6^), and bold black represent probiotic*s* (1 × 10^9^) + *C. sorokiniana* (1 × 10^9^), CFU/g. Data were analyzed by Kruskal-Wallis with subsequent Dunn’s test using GraphPad Prism 7.0.

HT-29 cells were infected with rotavirus Wa (MOI 0.01) and treated with of *L. plantarum* and *B. longum* (1 × 10^9^ CFU/mL each) metabolites and/or *C. sorokiniana* (1 × 10^9^ cells/mL). The microscopic cytopathic effect of rotavirus-infected cells treated with probiotic metabolites and *C. sorokiniana* was lower than that of infected cells without treatment. Lysates from metabolites-treated infected cells were used to evaluate the rotavirus viral titers (FFU/mL) in each assay (Figure 3). Rotavirus infectivity was reduced to 72, 52, and 64% after treatments with of *B. longum* and *L. plantarum* metabolites alone or in combination, respectively (Figure 4). Each probiotic and combination significantly reduced rotavirus infectivity (*p* < 0.05), with no significant difference between the use of *B. longum* or L. *plantarum*. After adding C. *sorokiniana* metabolites to each treatment, infectivity was significant reduced to 30, 8, and 5%, for *B. longum, L. plantarum*, or both probiotics, respectively (Figure 4). Notably, treatment with *C. sorokiniana* metabolites alone caused significant (*p* < 0.05) 4% reduction of rotavirus infectivity (Figure 4).

## Discussion

In this study, previously isolated microalgae were molecularly characterized by sequence homology and phylogeny of its 18S ribosomal RNA gene (Reyna-Martinez et al., 2018). The sequence was clustered with other sequences previously reported as *Chlorella sorokiniana* (Figure 1c). This microalga species has been used for wastewater treatment, biodiesel production, and as a traditional dietary supplement due to its antitumoral, antibacterial, and antiviral activities (Reyna-Martinez et al., 2018; Ciliberti et al., 2019; Hulst et al., 2019). The biochemical components of *C. sorokiniana* biomass grown under photoautotrophic conditions in our laboratory (Table 1), showed lower lipid, but higher protein, carbohydrate, and mineral levels than other microalgae such as *Chlorella vulgaris* cultured under photoautotrophic or mixotrophic conditions (Abreu et al., 2012; Dineshkumar et al., 2017).

In addition to *C. sorokiniana* characterization, its effect on probiotic viability was evaluated with *B. longum* and *L. plantarum* in flan (Figure 2). Among treatments, *L. plantarum* showed up and down numbers over time when supplemented with *C. sorokiniana*, both at 1 × 10^9^ cells/mL rate (Figure 2a). This observation may be related to population secondary successions. In this regard, nutrition source availability should be responsible, since other physicochemical factors did not change during the experiment. The results indicated that this microalga improved *L. plantarum* viability alone or in combination with *B. longum* (*p* < 0.05) (Figure 2). In this regard, previous studies with *C. sorokiniana* and *C. vulgaris* also showed prebiotic activity by enhancing *Lactobacillus rhamnosus* growth; in addition, the prebiotic effect of *C. sorokiniana* was higher than that of *C. vulgaris* (Lai et al., 2019). Other genera of *Chlorella* have shown potential for improving *L. acidophilus* and *B. lactis* viability in yogurt (Beheshtipour et al., 2012, 2013). Although in the present study the mechanisms underlying the improved viability of probiotics after the addition of microalgae were not addressed, these could involve microalgae production of substances responsible for the stimulatory properties from a nutritional factor, such as adenine, hypoxanthine, and free amino acids (Varga et al., 2002; Gyenis et al., 2005; Beheshtipour et al., 2013). In this study, improved probiotics growth by *C. sorokiniana* was observed with metabolically active microalgae. Our results demonstrated that *C. sorokiniana* might be used as an ingredient in dairy food products in order to improve the probiotics such as *B. longum* and *L. plantarum* shelf-life. This study supports other reports describing the potential use of microalgae and blue-algae biomasses in milk-based functional fermented feeds (Varga et al., 2002; Gyenis et al., 2005; Beheshtipour et al., 2013). Although *Chlorella* is not yet formerly defined as prebiotic, it was recently considered as a functional food. Indeed, *Chlorella* used as feeding supplement in pigs have shown gastrointestinal effect in the manage of mild digestive disorders and in the fecal microbiota (increasing *Lactobacillus* concentration and reducing *E. coli*) (Yan et al., 2012; Furbeyre et al., 2017). Following *in vitro* digestion and fermentation, *Chlorella* was observed to increase propionate-producing microorganisms population in the intestines, which was associated with postbiotic gut health (Jin et al., 2020). *Chlorella sorokiniana* postbiotic effects represent one of our further research interests.

In addition to the increased viability of probiotics by *Chlorella sorokiniana*, other studies with this microalgae genus demonstrated its antibacterial activity against intestinal pathogens (Beheshtipour et al., 2012; Lai et al., 2019). In this study, *B. longum* and/or *L. plantarum* plus *C. sorokiniana* antiviral activity was evaluated in rotavirus-infected HT-29 cells (Figures 3, 4). Probiotics/microalga and cell metabolites showed potential to reduce rotavirus cytopathic effect, however, assays were performed using only metabolites due to interferences of probiotics/microalga cells with the immunochemistry stain of rotavirus-infected cells in viral titration. Results described in Figure 4 indicated that rotavirus titers decreased in cell cultures treated with *B. longum* or *L. plantarum* metabolites or after treatment with both probiotics’ metabolites. Furthermore, the anti-rotavirus effect was improved after *C. sorokiniana* metabolites addition. Nevertheless, such improved anti-rotavirus effect cannot be fully attributed to the prebiotic properties of *C. sorokiniana*, since microalga metabolites showed a very potent antiviral effect *per se*, reducing rotavirus infectivity.

This study was designed to test the effect of *C. sorokiniana* on viability and antiviral activity of probiotics. Results showed an improved antiviral effect of probiotics, but unexpectedly, microalgae alone showed significant antiviral effect, which represents the first report on this matter to date. Recent reports showed *C. sorokiniana* effect against bacterial pathogens (Dineshkumar et al., 2017; Lai et al., 2019), whereas other microalgae species showed antiviral effects against murine cytomegalovirus (M-CMV) and herpes simplex virus type 1 (HSV-1) (Ibusuki and Minamishima, 1990; Shalaby, 2011; Santoyo et al., 2012). A recent study with *C. sorokiniana* demonstrated that the use of this microalga, before and after rotavirus infection in *in vitro* assays, increased type-I interferon expression levels, improving antiviral response and reducing the cytopathic rotavirus effect on HT-29 cells (Romero-Argüelles, 2020). Although *Bifidobacterium* spp. (Kim et al., 2014; Li et al., 2016; Olaya-Galan et al., 2016; Gonzalez-Ochoa et al., 2017) and *Lactobacillus* spp. (Ang et al., 2016; Olaya-Galan et al., 2016; Sunmola et al., 2019) antiviral effect, as well as the antimicrobial activity of *Chlorella* spp. have been documented, the present study showed that *C. sorokiniana* might be used as an ingredient in dairy food products to improve probiotics’ viability and their effect against pathogens. As far as we know this is the first *C. sorokiniana* report demonstrating antiviral effect; although, more studies are needed to confirm *C. sorokiniana* specific effect against rotavirus.

## Data Availability Statement

The sequence of microalga *Chlorella sorokiniana* (18S ribosomal RNA gen partial gene) analyzed in this study can be found in the GenBank database with the access number MN011866 (https://www.ncbi.nlm.nih.gov/nuccore/MN011866).

## Author Contributions

GG-O and PT-G conceived the study, designed, and supervised the experiments. SC-B and MD-G worked with the probiotics and microalga assays. IM-P, EA-U, and RG-F contributed with viral propagation and microtitration assays. NO contributed to growth experiments and biomass analysis. LF-M contributed in the sequence analysis and statistics. All the authors contributed to writing the manuscript, read, and approved the manuscript.

## Conflict of Interest

The authors declare that the research was conducted in the absence of any commercial or financial relationships that could be construed as a potential conflict of interest.
